# The role of Rapsyn in neuromuscular junction and congenital myasthenic syndrome

**DOI:** 10.17305/bb.2022.8641

**Published:** 2023-10-01

**Authors:** Xufeng Liao, Yingxing Wang, Xinsheng Lai, Shunqi Wang

**Affiliations:** 1 Institute of Life Science and School of Life Sciences, Nanchang University, Nanchang, China; 2 School of Basic Medical Sciences, Nanchang University, Nanchang, China

**Keywords:** Rapsyn, neuromuscular junction (NMJ), congenital myasthenic syndrome (CMS), mutation, medication, central nervous system (CNS)

## Abstract

Rapsyn, an intracellular scaffolding protein associated with the postsynaptic membranes in the neuromuscular junction (NMJ), is critical for nicotinic nerve receptor clustering and maintenance. Therefore, Rapsyn is essential to the NMJ formation and maintenance, and Rapsyn mutant is one of the reasons causing the pathogenies of the congenital myasthenic syndrome (CMS). In addition, there is little research on Rapsyn in the central nervous system (CNS). In this review, the role of Rapsyn in the NMJ formation and the mutation of Rapsyn leading to CMS will be reviewed separately and sequentially. Finally, the potential function of Rapsyn is prospected.

## Introduction

Rapsyn, as a receptor associate protein of the synapse, was first isolated from Torpedo electric organ and was named Torpedo 43K protein for the molecular weight [1]. Rapsyn was found as a major protein of nicotinic acetylcholine (nAChR) associated with the postsynaptic membranes of the neuromuscular junction (NMJ), being essential for nAChR clustering and maintaining [2]. In 1994, Rapsyn was early cloned and characterized from a mouse consisting of eight exons and extending over 12 Kb in the central region of chromosome 2 [3]. Human Rapsyn cDNA was first cloned in 1996, and the gene was mapped to the locus of chromosome 11p11.2–p11.1 [4].

**Figure 1.**
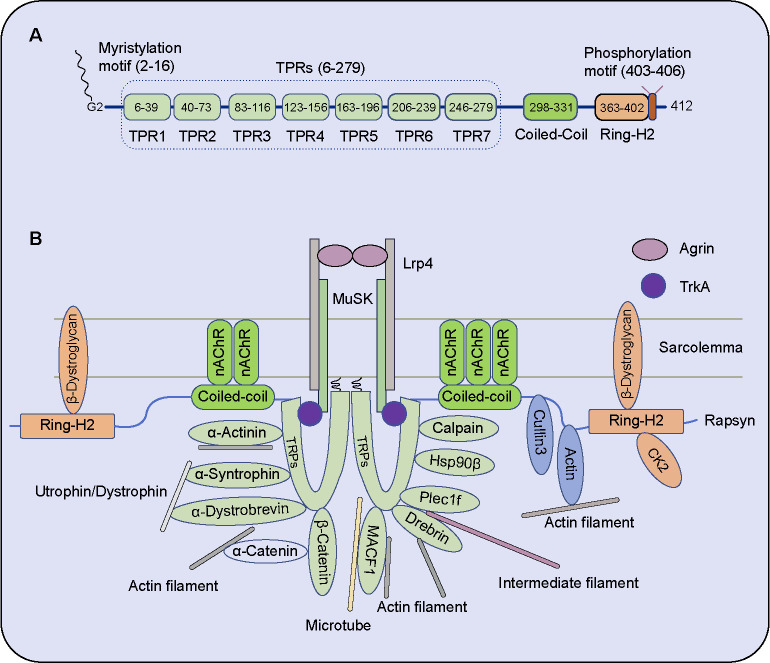
**Protein structural diagram and the interacting factors of Rapsyn.** (A) Rapsyn’s full length is 412 amino acids, and the mature protein’s first methionine is cut off. Rapsyn links to the postsynaptic membrane via an N-terminal myristoylated site (Gly2). Rapsyn contains a myristylation motif, seven tandem tetratricopeptide repeats (TPRs), a coiled-coil (CC) domain, a Ring-H2 domain, and a phosphorylation motif; (B) The factors interacting with Rapsyn in the NMJ. Most factors are associated with cellular skeletal protein, such as actin filament (F-actin), intermediated filament, and microtube; some form complex with Rapsyn. Rapsyn maintains the nAChR clusters via anchoring with sarcolemma or cellular skeletal protein directly/indirectly. CK2: Casein kinase 2; HSP90β: Heat shock protein 90β; Lrp4: Low-density lipoprotein receptor-related protein 4; MACF1: Microtubule actin cross-linking factor 1; MuSK: Muscle-specific kinase; nAChR: Nicotinic acetylcholine receptor; TPR: Tandem tetratricopeptide repeats; TrkA: Neurotrophic receptor tyrosine kinase 1; NMJ: Neuromuscular junction.

Rapsyn is tightly anchored in the postsynaptic membrane via an N-terminal myristoylated site (Gly2), followed by seven tandem tetratricopeptide repeats (TPRs), coiled-coil (CC) domain, and a C-terminal Ring-H2 domain (Figure 1A). Its N-terminal contains a consensus sequence subject to the covalent attachment of the myristate, and the immobilization in the cytoplasmic membrane face maintains a specialized network of the nAChR clusters in the NMJ of vertebrates [1]. TPRs are 34-amino-acid repeats forming two amphipathic α-helices, which regulate protein–protein interactions. CC domain is a large part, including a potential TPR motif. The C-terminal region is cysteine-rich and conforms to a zinc ring finger motif belonging to the Ring-H2 domain [5], which was identified to execute E3 ligase activity [6, 7].

Rapsyn is critical to the NMJ forming and maintaining, and Rapsyn mutant is one of the reasons causing the pathogenies of the congenital myasthenic syndrome (CMS). The role of Rapsyn in the NMJ formation and CMS will be reviewed separately and sequentially in the following sections. In addition, the potential function of Rapsyn in the CNS will also be summarized, which may give light on further research on Rapsyn.

## Rapsyn in the neuromuscular junction formation

The presynaptic substructure, postsynaptic substructure, and the surrounding Schwann cell compose the classic synaptic unit in the NMJ. The presynaptic motor nerve terminal can release its specialized versicles containing acetylcholine (ACh), and the ACh induces a contractile state of the muscle by regulating the electrical activity. The postsynaptic motor endplate is a tiny patch, occupying <0.1% of the muscle surface, and nAChRs aggregate into high-density clusters on the tiny size of the muscle cell membrane. Rapsyn is not only the specialized scaffold protein to be responsible for the postsynaptic motor endplate via anchoring nAChRs to the underlying cytoskeleton and the overlying basal lamina but also induces aggregating of nAChRs [8]. Rapsyn is not required to cluster the muscle-specific kinase (MuSK) in vivo [8].

The Rapsyn-deficient mice demonstrated the critical role of Rapsyn in 1995, and the mutant mice died of breath disability within a few hours of birth, resulting from the absent nAChR aggregate and abnormal nerve branching [3, 9]. In Rapsyn-deficient mice, MuSK remains concentrated at synaptic sites, but nAChRs fail to aggregate in the NMJ [10]. Moreover, Rapsyn is required for nAChR phosphorylation in MuSK signaling [10]. nAChRs localization is mediated by the motoneuron-derived Agrin and requires Rapsyn [11, 12]. Rapsyn interacts with nAChR via the α-helical motif between the α, β, and γ subunits [11]. Versatile factors interact with Rapsyn to induce and enhance nAChR clustering (Figure 1B).

### Overview function of Rapsyn domain

N-terminal 15-amino-acid of Rapsyn is sufficient to target green fluorescent protein (GFP) to the plasma membrane via myristylation. Two TPRs [1–90 amino acid (a.a.)] are sufficient to promote Rapsyn self-association, and the CC domain (298–331 a.a.) directly binds to the nAChR cluster [5, 13, 14].

The TPR domain of Rapsyn can interact with the bulk of the kinase domain, including MuSK, and the interaction between Rapsyn and MuSK is not dependent on the tyrosine phosphorylation of the MuSK sequences [15]. Intriguingly, MuSK cytoplasmic domain effectively co-distributes with Rapsyn, which confers TrkA (Neurotrophic receptor tyrosine kinase 1), an inactive receptor tyrosine kinase, to associate with Rapsyn [15]. Surprisingly, Rapsyn-induced MuSK clustering depends on the MuSK ectodomain but not the cytoplasmic domain, and the transmembrane protein β-dystroglycan may be the linker between MuSK extracellular domain and Rapsyn cytoplasmic domain [5].

### Association with nAChR

Neither the N-terminal myristylation nor the Ring-H2 domain of Rapsyn is required for stable contact with the postsynaptic membrane in the NMJ. The CC domain of Rapsyn is critical for nAChR aggregation [16]. nAChR is exceptionally responsive to nicotine. Independent of Agrin signaling, Rapsyn interacts with the loops of nAChR with different affinities via an α-helical structural motif, anchoring, and clustering highest for β-loop being followed by ɛ-loop and α-loop of nAChR [11]. Rapsyn construct lacks the α-helical domain resulting in severe alteration of nAChR turnover and synapse fragmentation [17].

Although Rapsyn and nAChR form an aggregate independent of neural Agrin, treating with neural Agrin leads to the pre-existing clusters between Rapsyn and nAChR separating directly, and new small clusters accumulate again [18]. The synaptic activity is dispensable for the Rapsyn insertion into the postsynaptic membrane of NMJ [16]. Intriguingly, in cultured myoblast without nAChR, Rapsyn mainly localizes to the lysosomes. However, nAChR can target the cell membrane without association with Rapsyn [16]. In the presence of nAChR, Rapsyn is localized to the membrane and induces the formation of nAChR clustering [16].

Rapsyn efficiently immobilizes nAChR. nAChRs are connected by three Rapsyn bridges at least to form a 2D network, and half of the nAChRs belong to Rapsyn-connected groups composed of 2–14 AChRs [19, 20]. Most nAChR is immobile, and 20% is confined to 50 nm. Devoid of Rapsyn, the immobile population of nAChR decreased three times, and half of the mobile nAChR restricted diffusion in domains of 120 nm. Surprisingly, the size of the nAChR cluster is strongly reduced with the presence of Rapsyn [21].

**Figure 2.**
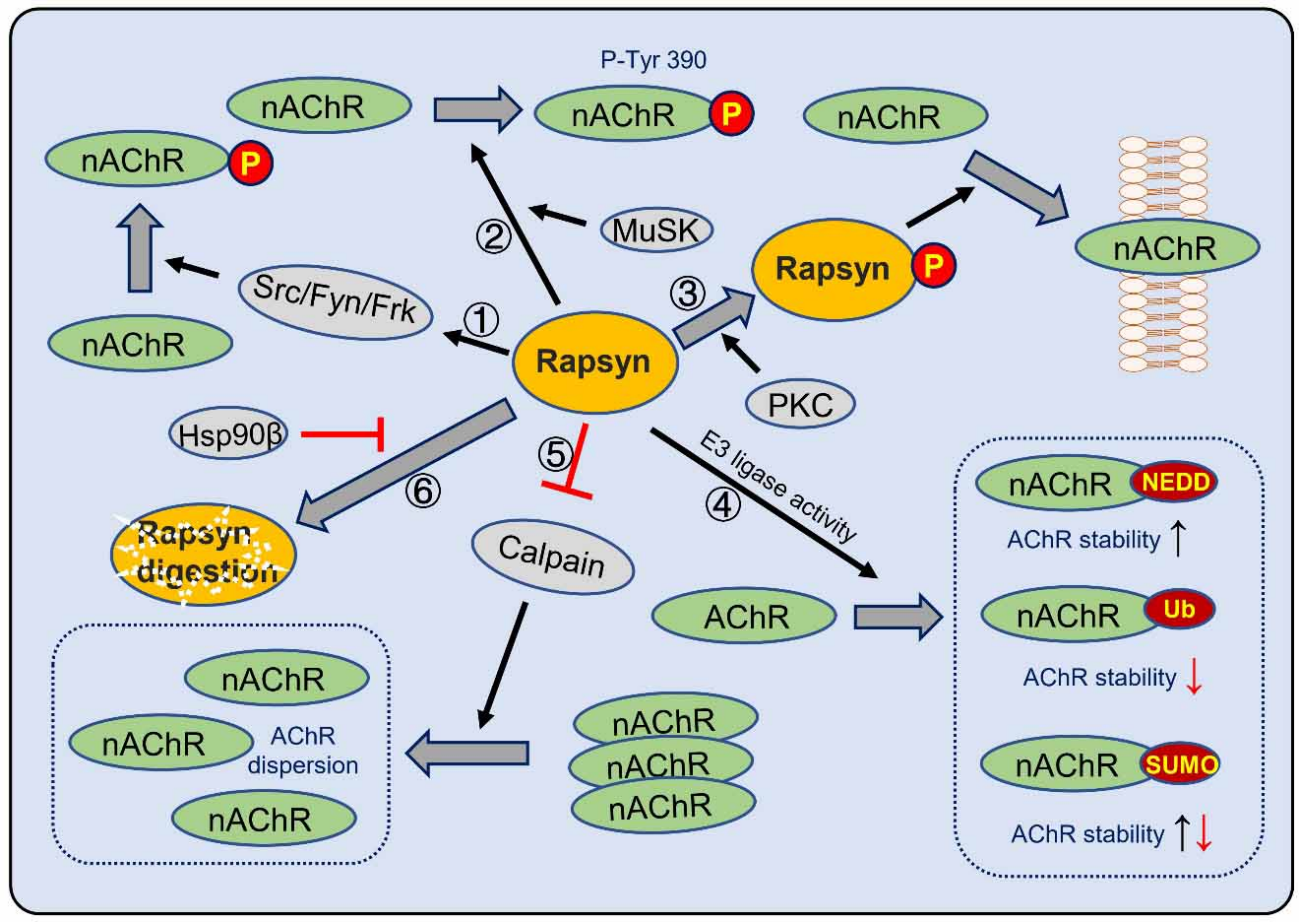
**Rapsyn-dependent signaling pathways in the NMJ end plate.** Rapsyn modifies nAChR in several ways, and its stability is also regulated by other factors. (1) Rapsyn can activate tyrosine kinases to phosphorylate nAChR; (2) Rapsyn induces the phosphorylation of nAChR β subunit Y390, and MuSK can enhance the phosphorylation; (3) Rapsyn may be phosphorylated by PKC and then induces nAChR insertion into the membrane; (4) E3 ligase activity of Rapsyn Ring-H2 domain modulates nAChR stability, including neddylation (NEDD), ubiquitination (Ub), and sumoylation (SUMO); (5) Calpain participates in acetylcholine-induced nAChR cluster dispersion, and Rapsyn inhibits its function; (6) HSP90β is necessary for Rapsyn stabilization (arrow means promoting, and vertical crossing lines in red color means inhibiting; “*↑*” indicates increase, and “↓” shows decrease). Frk: Fyn-related Src family tyrosine kinase; Fyn: FYN proto-oncogene, Src family tyrosine kinase; HSP90β: Heat shock protein 90β; MuSK: Muscle-specific kinase; NEDD: Neddylation; nAChR: Nicotinic acetylcholine receptor; P: Phosphorylation; PKC: Protein kinase C; Src: SRC proto-oncogene, non-receptor tyrosine kinase; SUMO: Sumoylation; Ub: Ubiquitination.

Rapsyn facilitates nAChR phosphorylation by localizing or activating tyrosine kinase via the C-terminal domain, which is sufficient and necessary for tyrosine kinase activation and tyrosine phosphorylation (Figure 2). MuSK alone cannot induce the phosphorylation of nAChR β subunit tyrosine 390 (Y390), but Rapsyn alone induces the phosphorylation of Y390. Furthermore, Rapsyn plus MuSK enhances the phosphorylation of Y390 more than Rapsyn alone, and deletion of the Rapsyn Ring-H2 domain abolishes the phosphorylation induced by MuSK [22]. The indirect activation of MuSK can promote Rapsyn-induced nAChR clustering, but the intermediate molecular between MuSK and Rapsyn is not well known [12].

The Ring-H2 domain of Rapsyn contains E3 ligase activity modulating nAChR (Figure 2). Modifications mediated by Rapsyn E3 ligase activity may include ubiquitination, neddylation, or sumoylation, which may affect the stability of the NMJ structure and function proteins [6, 23]. The mutation of cysteine 366 (p.C366A) abolishes its enzymatic function and severely impairs nAChR clustering [6, 12, 23].

Rapsyn interacts with Calpain, a calcium-dependent protease, suppressing Calpain protease activity (Figure 2). Calpain participates in ACh -induced nAChR cluster dispersion, and Rapsyn stabilizes nAChR aggregates by inhibiting the protease activity of Calpain [24].

### Association with skeleton protein and self-association

Rapsyn binds with Actin and Actinin as a scaffold protein in the NMJ [25, 26], bridging nAChR to the cytoskeleton and fixing the cluster. Agrin stimulates nAChR clustering and enhances the interaction of Rapsyn and α-Actinin [25, 26], which can be disrupted by cholinergic stimulation [26]. Downregulating expression of α-Actinin inhibits Agrin-mediated nAChR clustering [26].

The Ring-H2 domain of Rapsyn interacts with the cytoplasmic domain of β-Dystroglycan [13, 14, 27]. In Rapsyn-deficient mice, the nAChR cluster is absent in the postsynaptic endplate, and some peripheral membrane proteins are also missing, such as β-dystroglycan and Utrophin [27].

Rapsyn forms a complex with α-Syntrophin and α-Dystrobrevin at the crests of junctional folds in the NMJ. Like Rapsyn, α-syntrophin turnover is faster than nAChR [28]. Intriguingly, α-Syntrophin deficiency altogether abolishes the interaction between Rapsyn and α-Dystrobrevin in mice, which Utrophin can rescue. However, α-Dystrobrevin null does not affect the complex between Rapsyn and α-Syntrophin, nor the turnover of Rapsyn and α-Syntrophin [28].

The TPR domain of Rapsyn binds microtubule actin cross-linking factor 1 (MACF1), enhancing Rapsyn’s connection with microtubules and nAChR immobilization [25, 29–32]. MACF1 binds Rapsyn and serves as a synaptic organizer for the microtubule-associated proteins Microtubule-associated protein RP/EB family member 1 (EB1) and Microtubule-associated protein 1B (MAP1B) and the actin-associated protein Vinculin [29].

Rapsyn interacts with β-Catenin, and the latter is regarded as one of the linkers between the β-catenin-associated cytoskeleton and nAChR, independent T-cell factor (TCF) [33, 34]. Intriguingly, Wang et al. found that Wnt/β-catenin signaling negatively regulates nAChR cluster formation via repressing Rapsyn expression [35]. Wnt3a treatment inhibits Agrin-induced nAChR clustering and promotes nAChR cluster dispersion, which can be prevented by Dickkopf1 (DKK1), one of the antagonists of the Wnt/β-catenin signaling [35].

In addition, Rapsyn interacts with Plectin 1f (Plec1f) to bridge nAChRs and the intermediate filament network beneath the postsynaptic membrane [23, 36]. The interaction between Rapsyn and the cytoskeletal organizer can also be enhanced by Rapsyn binding Drebrin, an Actin, and microtubule cross-linker [31].

Each TPR of Rapsyn is composed of 34 amino acids and adopts a helix-turn–helix fold, which mediates dimerization and oligomerization [27, 37, 38]. Rapsyn forms clusters by oligomerization mediated by TRPs [39]. Substitution of a termination codon for Asp254 produced a truncated (28-kD) protein associated poorly with the cell membrane, and the mutant Rapsyn with TPRs 1–7 deletion fails to aggregate nAChRs [40]. No less than two TPRs are required for Rapsyn self-association [14].

### Phosphorylation on Rapsyn

Dependent on the thiamine (vitamin B1) triphosphate (ThTP) as the phosphate donor, histidine residue(s) of Rapsyn can be phosphorylated with endogenous protein kinase. In addition, Zn2+ inhibits phosphorylation [2, 41], indicating that the zinc finger domain controls the phosphorylation state of Rapsyn in the process [2]. However, the ThTP-dependent kinase has not been identified. Dephosphorylation of thiamine diphosphate (ThDP) and ThTP is coupled to the thiamine release upon electric stimulation of nerves. Thiamine co-release with ACh facilitates acetylcholinergic neurotransmission via interaction with taste 2 receptor member 1 (TAS2R1), activating synaptic ion currents [41].

Moreover, the zinc finger is also a consensus site for serine phosphorylation, but it is unclear whether the serine residue is phosphorylated or not [41]. Rapsyn associates with post-Golgi vesicles (PGV), one of the distal exocytic compartments, and is co-targeted explicitly with nAChR to the postsynaptic membrane. Rapsyn serine is not phosphorylated in the PGV, and Rapsyn serine and nAChR tyrosine phosphorylation occur in the postsynaptic membrane, which involves the regulation of cluster formation [42].

MuSK induces tyrosine phosphorylation of Rapsyn [11]. Rapsyn binds to each nAChR subunit via the intracellular loop between the receptor subunit’s third and fourth transmembrane segments [38], and nAChR directly interacts with Src-family tyrosine protein kinases. Rapsyn can activate tyrosine kinases, such as SRC proto-oncogene, non-receptor tyrosine kinase (Src), FYN proto-oncogene, Src family tyrosine kinase (Fyn), and fyn-related Src family tyrosine kinase (Frk), via forming a complex [43], and the complex phosphorylates nAChR β and δ subunits [43]. Moreover, APC regulator of WNT signaling pathway (APC) and Src-family kinases directly bind to nAChRs, stabilizing nAChRs [38].

Amino acids 403–406 of Rapsyn encompass a consensus sequence for protein kinase A (PKA) and protein kinase C (PKC) phosphorylation [13]. Rapsyn interacts with PKA type I via an amphipathic α-helical stretch (a.a. 299–331) of the Rapsyn CC domain, indicating Rapsyn anchors PKA type I [17, 44, 45]. The inhibitor of PKC and the broad-spectrum kinase inhibitor staurosporine abrogate nAChR insertion into the membrane without affecting the insertion of Rapsyn (Figure 2). Moreover, the insertion of Rapsyn or nAChR is not disturbed by PKA inhibitors [18]. The insertion of Rapsyn and nAChR may be operating independently.

Rapsyn has strong interaction with protein kinase Casein kinase 2 (CK2), but the potential role for the CK2/Rapsyn interaction is unclear because Rapsyn is not phosphorylated by CK2 [46].

### Transportation and turnover

Rapsyn undergoes liquid–liquid phase separation (LLPS) and condensates into liquid-like assemblies [20], which can recruit AChRs and signaling proteins for postsynaptic differentiation to form membrane compartments. Multivalent binding of TPRs is essential to Rapsyn LLPS [20, 47].

Rapsyn interacts with heat shock protein 90β (HSP90β), and disruption of the interaction attenuates nAChR cluster formation in vitro and impairs the development and maintenance of NMJ. HSP90β is necessary for Rapsyn stabilization and mediates the proteasome-dependent degradation, indicating HSP90β regulates Rapsyn turnover [48].

The Rapsyn/nAChR interaction leads to nAChR clustering and a clustering-independent fast recovery from desensitization [49]. The halftime of Rapsyn recovery at clusters is about 1.5 h, whose turnover is 3–6 times quicker than nAChR [18, 48, 50], implying Rapsyn may be a bi-functional molecular as both an adaptor protein and a signaling protein [23]. In addition, nAChR turnover is sensitive to the alteration of the synaptic activity, whereas Rapsyn is unaffected, illustrating distinct mechanisms of turning over between them [50].

Rapsyn mediates nAChR clustering and maintenance by interacting via lipid rafts [51]. Rapsyn and AChR are co-transported in the same PGV to the innervated surface of the Torpedo electrocyte [42, 52]. After being co-transfected into COS-7 cells, Rapsyn and AChR co-distribute within distal exocytic routes besides at the plasma membrane [53]. Rapsyn, an itinerant vesicular protein in the lipid rafts, may play a dynamic role in sorting and targeting its companion receptor to the postsynaptic membrane [53, 54]. Rapsyn cannot form self-clusters separating from nAChRs before synapse formation in zebrafish. Without nAChR, Rapsyn is retained in the Golgi complex in the postsynaptic cell of zebrafish NMJ [55].

### Change of Rapsyn expression

In aging mice, denervated fibers accumulate due to reinnervation failure, and nAChR cluster density negatively correlates with endplate Rapsyn [56]. Silencing Rapsyn expression with short hairpin RNA (shRNA) in the NMJ causes a one-third reduction in the protein level of Rapsyn and nAChR, but it leads to the sodium channel protein being increased two third. Unexpectedly, secondary folds of the endplate in the Rapsyn-silencing muscle increase, and the neuromuscular transmission are mildly damaged [57].

Skeletal muscle undergoes repeating cycles of denervation and reinnervation in adult life, and in very advanced age, the denervated muscle fibers accumulate remarkably, accompanied by severe muscle atrophy impairing mobility. Denervated myofibers in senescent rats (36 months) muscle are on 35%–50% smaller than innervated fibers in the young adult rat (8–10 months) [58]. Impaired capacity reinnervation might contribute to the accumulation of persistently denervated muscle fibers in the normal process of aging muscle [59]. Rapsyn expression is a benefit to the AChR intensity in aging muscle. Compared to the young rat (8 months) muscle, Rapsyn at the endplate in the very old rat (35 months) muscle increases with only a 10% decline in AChR intensity. However, in the Sarco mice (8 months) muscle, a murine model of sporadic denervation, Rapsyn expression declined to associate with AChR intensity decrease by 25%, although transcripts of AChR subunits are upregulated [59]. Lamin A/C, an intermediate filament factor, is decreased in the aging skeletal muscle of mice, its deficient in skeletal muscle (HSA-Lamin -/-) causes NMJ deficits, including progressive denervation, AChR fragmentation, and neuromuscular dysfunction, and the NMJ deficits can be attenuated by expression of Rapsyn in muscles [60]. The very old rat muscle exhibits much more accumulation of small fibers (>20%), a sign of persistent sporadic denervation, than small fibers in Sarco mice (<6%), suggesting a reduced capacity of reinnervation in aging muscle [59].

The mechanisms of NMJ decline in aging animals are not clear. NMJ fragmentation is associated with the aging process and could result from the degeneration and regeneration of muscle fiber segments [23, 61–64]. However, NMJ fragmentation per se does not imply a decline in fundamental features of transmission because neuromuscular transmission at the highly fragmented NMJ in the very old mice (26–28 months) diaphragms is not weakening, compared to in the middle-aged mice (12–14 months) [65].

### Special exception of Rapsyn in nAChR clustering and Rapsyn in zebrafish

Rapsyn is dispensable for nAChRs clustering in the superior cervical ganglion (SCG), one of the typical cholinergic synapses in mammalian sympathetic ganglions [66]. Firstly, although Rapsyn RNA is readily capable of being detected in the SCG, Rapsyn protein is undetectable at the nAChR clusters. Secondly, Rapsyn can form clusters with neuronal AChR or muscle AChRs in heterologous cells, but only the last clusters appear on the plasma membrane. Neuronal AChR clusters induced by Rapsyn are always intracellular. Lastly, in Rapsyn-deficient mice, both non-synaptic and synaptic AChR clusters can form without Rapsyn [66]. Therefore, the evidence indicates that Rapsyn is not an essential mediator of nAChR clustering at SCG synapses.

A Rapsyn-deficient mutant line of zebrafish shows fatigue. The mutant zebrafish exhibits exaggerated depression in response to high-frequency stimulation than the wild-type. Moreover, the vesicle reloading and release in the mutant zebrafish is significantly slower at individual release sites during high-frequency activities. Accordingly, compromised presynaptic release and reductions of postsynaptic receptor density in the mutant zebrafish collectively decrease synaptic strength, thus causing use-dependent fatigue [67].

### Promoter of Rapsyn

According to one synapse-specific transcription model, the N-box, with CCGGAA as the core consensus sequence, is required for mediating transcription in NMJ subsynaptic nuclei. Moreover, N-box confers transcription of nAChR, Utrophin, and ACh esterase genes [68–71]. Although Rapsyn is expressed explicitly in the postsynaptic membrane in the NMJ, N-box is not found in the Rapsyn promotor.

There are two Kaiso sites and three E-box in the Rapsyn promotor region, and one Kaiso site partially overlaps one of the E-box motifs (E-box-Kaiso site) [68, 72]. Kaiso belongs to the POZ, a zinc finger family transcription factor, and the specific core consensus sequence is CTGCNA (N is any nucleotide). δ-catenin is one of the binding partners for Kaiso and forms a complex with Kaiso. Rapsyn interacts with Kaiso, and its promotor can be activated by Kaiso and δ-catenin, strongly indicating that Rapsyn is one direct sequence-specific target of Kaiso and δ-catenin [68].

### Ubiquitylation and other function

A post-translational mechanism regulates Rapsyn protein stability. 43 kDa receptor-associated protein of the synapse homolog (rpy-1) in Caenorhabditis elegans is a Rapsyn homolog in mammals, ubiquitinated by a similar complex in RPY1 and RAPSYN [73].

The Ring domain of Rapsyn comprises E3 ligase function, and the enzymatic activity via mediating nAChR ubiquitylation/neddylation is essential for nAChR clustering [6]. Mutation of cysteine 366 (p.C366A) in the zinc finger abolished its enzymatic function.

Recently, research found that Rapsyn can be co-immunostaining with chromodomain helicase DNA-binding protein 8 (CHD8) at the sarcoplasmic side in the NMJ, and the potential role of the interaction is also unclear [74]. Rapsyn overexpression in muscles attenuates the NMJ deficits in Lanim A/C deficient mice [75].

### Congenital myasthenic syndrome due to Rapsyn mutation

Myasthenia gravis (MG) is an autoimmune disease disabling NMJ function and causing fatigable ocular, bulbar, and limb muscle weakness. CMS has a similar phenotype, but it belongs to a genetic disease with an onset early in life due to mutations of the NMJ structure gene or NMJ function proteins [6, 76–82]. In most patients, CMS clinic symptoms present at birth or infancy, seldom present in the second or third decade [83]. More than 35 different genes with mutation cause CMS. Mutations of genes for postsynaptic development or function are dominant in CMS cases, and among them, Rapsyn mutations and nAChR subunits malfunction are prevalent [78–80].

**Figure 3.**
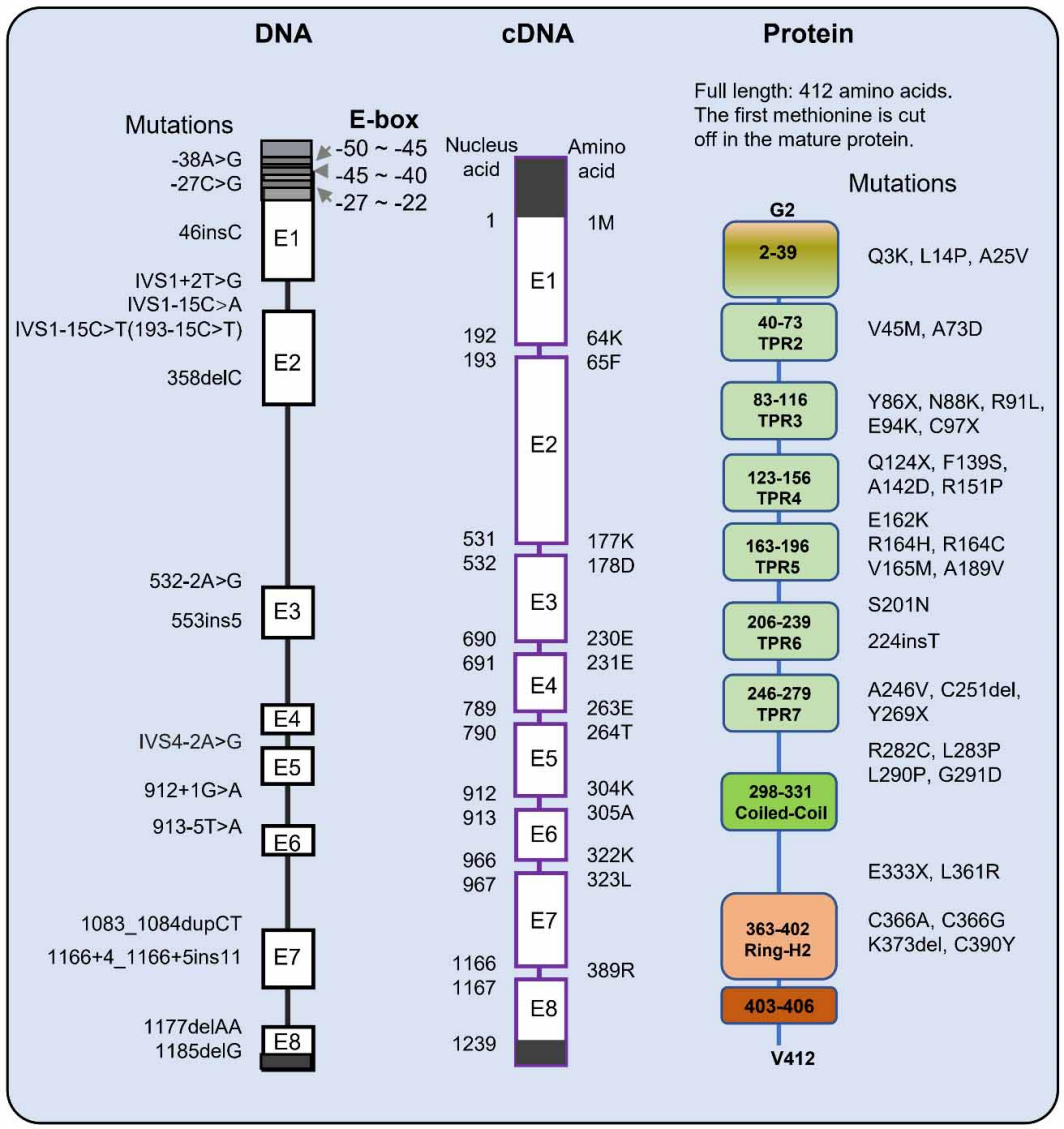
**Mutations of Rapsyn in congenital myasthenic syndrome.** Missense mutation or nonsense mutation of Rapsyn is shown in the right panel, and the “X” in the mutation represents nonsense mutation. Insertion and deletion in exon or intron causing frameshift of Rapsyn are shown in the left panel. The number in the left panel represents the nucleus acid number in the cDNA, and the number in the right panel means the amino acid number at the protein level.

Molecular mechanisms of Rapsyn regulating NMJ formation have made much progress, and Rapsyn mutation accounts for about 14%–27% of CMS cases [77, 81, 84–91]. Over 50 mutations are dispersed through the entire RAPSYN gene, associating with CMS [92, 93]. Versatile mutations of Rapsyn causing CMS are shown in Figure 3.

Different mutant Rapsyn reduces Rapsyn expression, impedes Rapsyn self-association, hinders Rapsyn colocalization with nAChR, and impairs nAChR clustering in the last [76–78]. Rapsyn mutant-causing CMS is reviewed in this section, mainly in the order of the mutant region.

### E-box mutation

E-box consensus sequence CANNTG (N is any nucleotide) is bounded by myogenic determination factors governing the specification and differentiation of muscle cells. Three consecutive putative E-box consensuses near the codon 1 were identified in the promotor region of Rapsyn, locating at −27 to −22 as CAGCTG, −40 to −35 as CAACTG, and −50 to −45 as CATGTG [68, 94]. E-box mutation in the Rapsyn promoter region causes CMS via downregulation of Rapsyn and endplate nAChR deficiency in the NMJ [94].

One patient is heterozygous for p.N88K and −27C>G in the Rapsyn promotor, which may be the critical reason for the failure of Rapsyn transcription [94]. In luciferase reporter assay, −27C>G attenuates reporter gene expression in C2C12 myotubes and myogenic differentiation 1 (MyoD) or Myogenin-transfected HEK cells and impairs the enhancer activity of SV40 promoter [94].

Seven patients carry homozygous −38A>G in Rapsyn promotor without changing the E-box consensus sequence CANNTG [94, 95]. However, −38A>G also attenuates reporter gene expression in luciferase assays [94]. Moreover, −38A>G localizes in the overlap region of the Kaiso site and E-box motifs (E-box-Kaiso site) [68, 72, 96], and the mutation may also affect the activation from Kaiso and δ-catenin.

### TPR domain

The TPR domain of Rapsyn mediates its dimerization and oligomerization, and at least two TPRs are required for Rapsyn self-association [14, 39]. Nearly half of Rapsyn mutations causing CMS are located in the TPR domain. Theoretically, a mutation in the TRP is regarded as impairing Rapsyn-inducing nAChRs clustering via reducing Rapsyn self-association.

p.L14P not only localizes in the TPR1 domain of Rapsyn of Rapsyn but also changes the amino acid in the myristylation motif. Accordingly, it predicts potential conformational change at the Rapsyn N-terminal membrane association [83, 96–98]. p.A25V [99] of Rapsyn impairs the association of Rapsyn with nAChR and prevents nAChR clustering [100]. p.A25V and p.L14P in TPR1 may disrupt the interaction of Rapsyn and nAChR or impairs binding to plasm membrane via affecting myristylation.

In the TPR2, p.V45M (c.133 G>A) of Rapsyn is unable to co-cluster with nAChRs [101]. Mutation c.46insC of Rapsyn predicts truncation of the protein [83, 97, 98], leading to TPR1 and TPR2 deficiency. Therefore, the mutation abolishes Rapsyn dimer formation, which is also one of the bases for nAChR clustering, so it is not surprising to cause a severe effect in the CMS. p.A73D (c.218C>A) is the last amino acid residue mutation in TRP2 and causes CMS [102].

TPR3 mutation causing CMS includes several sites, such as p.Y86X [103], p.N88K [83, 86, 94, 96, 97, 100, 104–120], p.R91L [83, 99, 105], p.E94K [116], and p.C97X [121]. The functional effects of Rapsyn mutations do not always correlate with the proposed function of the mutant domain [100]. For instance, the TPR domain regulates protein–protein interaction [122], affecting the Rapsyn association. However, p.N88K has some different effects. The number of Agrin-induced nAChR clusters incorporating p.N88K of Rapsyn is decreased by 30%, and accompanied by withdrawing Agrin, the cluster number decreases severely, suggesting the clusters comprising Rapsyn mutant p.N88K are unstable [100].

p.N88K (c.264C>A) of Rapsyn, one of the most prevalent in the European CMS cohort, does not affect Rapsyn self-association but hinders Rapsyn recruitment nAChR clustering [83, 86, 94, 96, 97, 104–109]. p.N88K of Rapsyn is homozygous or heterozygous with other types of Rapsyn mutation in the patients. p.N88K homozygous patients were usually mildly affected, even with no symptoms [79, 86, 97, 109]. p.N88K is regarded as an ancient founder mutation [83, 108, 123, 124], and surprisingly, it is not infrequent in the healthy population, with five heterozygous p.N88K out of 300 healthy controls [108].

Homozygous p.N88K Rapsyn mutation impairs Rapsyn and nAChR aggregation, and some patients, due to homozygous p.N88K endure mild limb and ocular weakness without bulbar and respiratory dysfunction [125]. Strikingly, mutant mice with p.N88K homozygous Rapsyn die soon at birth with severe NMJ deficits [126]. Tyrosine phosphorylation of Rapsyn is required for the self-association and ligase activity. p.N88K mutation inhibited Rapsyn E3 ligase activity by reducing its tyrosine phosphorylation and self-association [126].

The mutation c.358delC (p.Q120Sfs*8) [116] of Rapsyn leading to frameshift does not cause severe symptoms, which may be due to carrying the other mutation p.N88K compensating Rapsyn function, and the phenotype is not distinguishable in the clinic [86]. However, heterozygous for p.N88K and p.Q124X, resulting in the truncation of Rapsyn [83], causes the pathogenesis of CMS with severe symptoms. The missense mutation p.F139S (c.416T>C) [127], p.A142D, and p.R151P [121] in the Rapsyn TPR4 domain are also related to CMS.

p.E162K (c.284G>A) [128, 129] is belonged to the linker between TPR4 and TPR5 of Rapsyn, endowing the inability of Rapsyn co-clustering with nAChRs [101]. p.R164C (c.490C>T) [118, 130], p.R164H (c.491G>A) [131], p.V165M (c.493G>A) [86, 108, 115, 116, 132], and p.A189V (c.566C>T) [127] in Rapsyn TPR5 domain are the common missense mutations causing CMS, which does not hider Rapsyn self-association, but diminishes co-clustering of Rapsyn with AChR. The mutation 553insCTGTT (553ins5) brings frameshifts and truncation of Rapsyn and leads to CMS [83, 98]. However, the pathogenicity of mutation p.S201D between Rapsyn TPR5 and TPR6 domain causing CMS is uncertain [128]. And p.S208R (c.624C>G) missense mutation in the Rapsyn TPR6 domain may weaken Rapsyn self-association [133].

The mutation p.224insT (c.673_676insACT) of Rapsyn indicates to abridge of the coiled string connection between the loops in the TPR6 domain, which may prevent protein looping and consequently impair Rapsyn self-association or Rapsyn co-clustering nAChR [95]. p.A246V (c.737C>T) [134] and p.C251del (c.752_754delGCT) [135] bring missense mutation to the beginning of Rapsyn TRP7 [134]. p.Y269X of Rapsyn predicts truncation of the protein in the Rapsyn TPR7 domain without the CC domain and Ring-H2 domain of Rapsyn, bringing severe consequences [97, 98]. The missense mutation p.R282C (c.844C>T) [135], p.L283P [130], and p.L290P (c.869T>C) [86, 116], between the TRP7 and CC domain of Rapsyn, does not affect Rapsyn self-association but impairs Rapsyn co-clustering with AChR.

### C-terminal of Rapsyn

There are several types of mutation causing CMS in the Rapsyn CC domain, such as frameshift mutation 1177delAA in the Ring-H2 domain [97, 98, 110] and a large deletion of about 4.5 kb after exon6 (in the CC domain) [124].

The nonsense mutation p.E333X truncates Rapsyn before the Ring-H2 domain [136]. p.L361R [99] is located between the CC and the Ring-H2 domain of Rapsyn [83, 100]. p.L361R reduces Rapsyn level via decreasing expression or increasing turnover of Rapsyn, which reduces Rapsyn colocalization with nAChR and dramatically impairs the stability of nAChR clusters [100]. The number of Agrin-induced nAChR cluster incorporating mutant Rapsyn is reduced by 60%, and withdrawing Agrin, the cluster number decrease severely, indicating the unstable of the clusters similar to the part function of N88K [100].

Mutation in the Rapsyn Ring-H2 domain is not rare, and a partial deletion of the Rapsyn Ring-H2 domain leads to premature termination of pregnancy [137]. The mutant fetuses displayed no respiratory movement and fixed limb positions [137]. Besides Rapsyn being linked to β-Dystroglycan via the Ring-H2 domain, the domain exhibits E3 ligase activity. p.C366A and p.C366G [117] prove that the cysteine residue is necessary for the ligase function, and the mutation impairs nAChR clustering in vitro and in vivo. p.K373del of Rapsyn may affect Ring-H2 function, leading to CMS [99].

### Intron mutation and frameshift mutation of Rapsyn

The c.913-5T>A mutation of Rapsyn leads to the transcription skipping exon 5 [138]. In CMS patients, the mutations of Rapsyn c.532-2A>G [119] and c.912+1G>A [133] may also change the splice site in a similar mechanism.

The frameshift mutation (1083_1084dupCT in exon7, p.Y362Sfs*10) of Rapsyn lies in the Ring-H2 domain [103, 108, 139]. The duplication causes the reading-frame disruption during translation, and premature termination at codon 371, which leads to E3 ligase activity is abolished. 1177delAA causes a frameshift mutation in the Rapsyn Ring-H2 domain, and the predicting 82 missense codons plus one stop codon disrupts the Ring-H2 domain [136]. p.K373del causes rapid degradation of the mutant [100].

In addition, the patient with Rapsyn heterozygous mutation c.1166+4_1166+5insAAGCCCACCAC (c.1166+4_1166+5ins11) [134] in RAPSYN, which is identified as skipping exon7 in transcription by RT-PCR [134]. Although the mutation c.1185delG (p.T396Pfs*12) [116] leads to the frameshift of Rapsyn, it does not cause more severe symptoms in the clinic [86].

**Figure 4.**
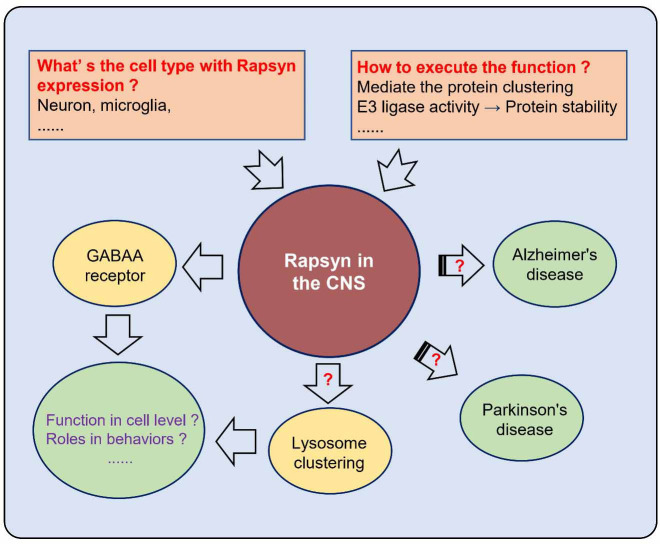
**Schematic diagram of the potential role of Rapsyn in the central nervous system.** The first important question in the research of Rapsyn’s role in the CNS is what is the cell type expressing Rapsyn. It is unknown whether the Rapsyn function in the CNS is similar to the role in the neuromuscular junction, such as mediating protein clustering, lysosome clustering, and E3 ligase activity. CNS: Central nervous system.

Intron mutation may lead to a change in mRNA splice and maturation. The splice mutation (IVS1-15C>A) of Rapsyn changing in the intron 1 generates a novel acceptor splice site (CAG/TCGCTG), causing 13 nucleotides of the intron retention in the mature mRNA and subsequently leads to a frameshift transcript, which produces a termination codon in exon 2 after 96 missense amino acids [130].

The splice site mutation IVS4-2A>G is an acceptor splice site mutation during mRNA maturation, foreshadowing a splicing error [103, 108]. The mutation IVS1+2T>G [111] and IVS1-15C>T [85] in Rapsyn may be owing to splice site change in Rapsyn causing CMS.

### Medication

CMS patients due to Rapsyn remarkably respond to anticholinesterase medication [78, 79, 83, 86, 90, 91, 100, 102–104, 106, 134, 136, 140], and CMS patients with Rapsyn mutation can be improved during early childhood, experience some episodic crises precipitated by the minor infections, and usually are resolved around age 6. After this challenging period, minimally symptomatic remains. Pyridostigmine, an acetylcholinesterase inhibitor, is the most commonly used CMS treatment [81]. Medication on CMS due to Rapsyn mutation is beneficial from the treatment with pyridostigmine, and sometimes, addition with 3,4-diaminopyridine (3,4-DAP) [91, 141], ephedrine [132], or albuterol results in significant clinical improvement [76–79, 87, 100, 102–104, 106, 134, 136]. Exceptions occasionally occur [142]; G.O. Skeie found that CMS patients were not responding due to homozygous p.N88K of Rapsyn to acetylcholinesterase inhibitors [143].

Fluoxetine, a selective serotonin reuptake inhibitor, is a blocker for nAChR long-lived open channel and is used to treat slow-channel CMS. Fluoxetine worsens clinically and electro-physiologically the phenotype of a CMS case due to p.N88K homozygous of Rapsyn [106, 142, 144]. The patient’s clinical and electrophysiological phenotype is improved via the introduction of pyridostigmine [106, 142, 144].

## Potential function of Rapsyn in the central nervous system

Rapsyn is expressed in muscle and nonmuscle cells and is tightly associated with the cytoplasmic membranes, demonstrating that Rapsyn is not specific to skeletal muscle-derived cells [145]. Therefore, Rapsyn executes its function unrelating with nAChR clustering [145]. Rapsyn can induce GABAA receptor or AchR clustering, implying that Rapsyn may have a role in the CNS [5, 146, 147]. The potential functions of Rapsyn in the CNS are summarized in Figure 4.

However, it was reported that Rapsyn has little expression in the brain. The reason lies that less than 20% of Genes with RPKM-value above 1 in all samples were discarded in the BrainScope. Based the brain-map.org, Vergoossen et al. found that Rapsyn is expressed in many areas in the brain, such as the hypothalamus, basal forebrain, amygdala, parahippocampal gyrus, cingulate gyrus, white matter, mesencephalon, pons, and myelencephalon [84].

In cells devoid of nAChRs, Rapsyn specifically induces lysosome clustering at a high density in the juxtanuclear region but does not affect other intracellular organelles distribution. In Rapsyn-deficient myoblasts, lysosomes are highly dynamic and scatter within the cell, leading to an increase in lysosomal exocytosis [148]. Furthermore, the E3 ligase activity of Rapsyn may play a critical function in the CNS.

In addition, based on the meta-analysis in different populations, Rapsyn is found to associate with the pathogenesis of Alzheimer’s disease (AD) [149, 150], Parkinson’s disease (PD) [151], and lacunar stroke [152].

## Conclusion

Rapsyn is a critical protein in the NMJ formation and maintenance via inducing and maintaining nAChR clustering. Therefore, the Rapsyn mutant has severe effects on NMJ function, which is one of the reasons causing CMS pathogenies. Fortunately, patients with CMS due to Rapsyn mutation remarkably respond to anticholinesterase medication, such as pyridostigmine, and sometimes, addition with 3,4-DAP, ephedrine, or albuterol results in significant clinical improvement. Research implies that Rapsyn may exhibit some roles in the CNS, and the known molecular mechanism of Rapsyn in the PNS may give a clue to explore Rapsyn action mode in the CNS.
